# Tirofiban: A Rare Cause of Thrombocytopenia in a Patient Undergoing Percutaneous Coronary Intervention

**DOI:** 10.7759/cureus.18217

**Published:** 2021-09-23

**Authors:** Amit Gulati, Aparna Tiwari, Vijay Shetty, Ifeanyi Nwosu, Sakshi Khurana

**Affiliations:** 1 Internal Medicine, Maimonides Medical Center, Brooklyn, USA; 2 Radiology, New York Presbyterian-Columbia University Irving Medical Center, New York City, USA

**Keywords:** intervention, coronary, percutaneous, thrombocytopenia, tirofiban

## Abstract

Patients admitted to the hospital can develop thrombocytopenia due to multifactorial causes. It can be pseudo-thrombocytopenia or true thrombocytopenia. Among patients admitted for chest pain, coronary angiography (CAG) is a common diagnostic test to evaluate patients for coronary artery disease (CAD). Normally, patients undergoing angiogram receive antiplatelets and anticoagulants pre-catheterization, and platelet aggregation inhibitor agents are sometimes used during and after CAG like in patients with high thrombus burden. Glycoprotein IIb/IIIa receptor inhibitors are a type of platelet antiaggregant agents that can cause severe thrombocytopenia in few cases.

We present a case of a 68-year-old patient who came to the emergency department with inferior wall ST-segment elevation myocardial infarction and underwent angiography and had percutaneous coronary intervention (PCI) done. He was administered tirofiban during the angiogram that caused acute severe thrombocytopenia decreasing platelets count to 4000/microliter within one day. Patients' platelets gradually recovered after platelets transfusion.

## Introduction

With the advent of technology, coronary angiography (CAG) and PCI are common investigations to evaluate patients for coronary artery disease (CAD). Patients undergoing angiogram receive antiplatelets and anticoagulants, and platelet aggregation inhibitor agents are reserved for high-risk patients like those with a high thrombus burden on coronary angiogram. Glycoprotein IIb/IIIa receptor inhibitors are a type of platelet antiaggregant agents that can cause severe thrombocytopenia in very few cases.

Glycoprotein IIb/IIIa receptors are present on the membranes of the platelet and are responsible for platelet aggregation. It does so by crosslinking the von Willebrand factor and fibrinogen. These are one of the major targets in the management of patients with the acute coronary syndrome (ACS). Tirofiban is a small, non-peptide, specific, and competitive glycoprotein IIb/IIIa inhibitor (GPI), and when administered intravenously, it inhibits platelet aggregation in a concentration-dependent manner. It can cause severe thrombocytopenia, the pathogenesis of which is likely due to drug-dependent antibodies that attach to platelets only in the presence of the drug. Severe thrombocytopenia can set in immediately after exposure to tirofiban [1].

We present a case of a 68-year-old patient who presented with inferior wall ST-segment elevation myocardial infarction (STEMI), who during the course of hospital stay for ACS developed severe thrombocytopenia.

## Case presentation

**Figure 1:**
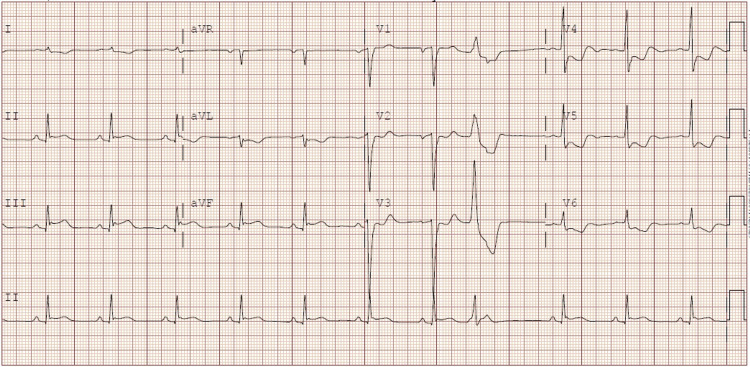
Electrocardiogram of the patient with ST-segment elevation in the inferior leads (lead II, III, aVF). There is downsloping ST-segment depression with T-wave inversion in V4-V6 with isolated PVC in lead V3 as well. PVC: premature ventricular contraction; aVF: augmented voltage foot

A 68-year-old Hispanic man with a history of coronary artery disease (STEMI four years prior to presentation) status post-bare-metal stent placement and unclear compliance with medications, active smoker presented with mid sternal chest pain that started early morning on the day of presentation. In the emergency department (ED), his temperature was 97.8°F, blood pressure was 108/53mmHg, respiratory rate was 24 per minute, heart rate was 95/minute, and his oxygen saturation on pulse oximeter was 98% on room air. On examination, he was a healthy-looking man in mild discomfort. On cardiac examination, no murmurs, rubs, or gallops were heard. Electrocardiogram showed ST-segment elevation in leads II, III, augmented voltage foot (aVF), ST-segment depression, and T wave inversion in leads V4-V6 and premature ventricular contraction in lead V3 (Figure 1).

Troponins were 0.08 ng/ml. He was loaded with aspirin 325 mg and ticagrelor 180 mg. He was also started on heparin drip and was taken emergently to the cardiac cath lab for percutaneous coronary intervention (PCI). He was found to have 100% stenosis in left circumflex artery with a large thrombus and a drug-eluting stent was placed. The patient was admitted to the cardiac critical unit for further monitoring. He was started on tirofiban as there was a large thrombus burden. Blood work repeated 8 h after the procedure showed thrombocytopenia with platelet count reduced from 208,000 to 4000 per microliter, with no significant change in white blood cell count and hemoglobin. The patient did not endorse any chest pain, bleeding, or shortness of breath. On physical examination, there were ecchymoses over dorsal aspect of right hand and right wrist along with a few scattered ecchymoses over the chest were noted.

On review of peripheral smear, manual platelet count was 10,000 per microliter, with normochromic normocytic red blood cell and normal white blood cell. No schistocytes were seen. Hematology was consulted for a sudden severe decline in his platelet count to 4000/microliter within a few hours. Other lab works including prothrombin time (PT), partial thromboplastin time (PTT), fibrinogen, and D-dimer were normal. Heparin-induced thrombocytopenia (HIT) platelet antibody tested negative. Haptoglobin, reticulocyte count, and lactate dehydrogenase (LDH) were also normal. Heparin and tirofiban drip were stopped and the patient was transfused one unit of single donor platelets. Aspirin and ticagrelor were continued. Soon after stopping the tirofiban drip and after the platelet transfusion, his platelet count improved to 56,000/microliter (within a span of four hours) and continued to rise to 77,000/microliter in 72 hours. At that point, the patient was discharged and a follow-up appointment was scheduled.

## Discussion

In critically ill patients admitted to medical wards or cardiac critical care unit, thrombocytopenia is one of the most common hemostatic disorders [2]. The etiology for thrombocytopenia can be multifactorial but generally, it could be attributed to reduced production of platelets, enhanced consumption, or secondary to hemodilution. Sometimes an unexplained low platelet count could be due to laboratory artifact, known as pseudo-thrombocytopenia [3].

Patients undergoing coronary angiogram (CAG) or angioplasty are given heparin during the procedure to prevent thrombosis, especially if the procedure is complicated and long. Similarly, the critically ill patients, in medical or surgical intensive care units (ICU), are at high risk of deep vein thrombosis (DVT) or pulmonary embolism (PE) and are given heparin, thus heparin-induced thrombocytopenia (HIT) is one of the most commonly suspected etiology of thrombocytopenia, although it is rarely proven [4]. Critically ill patients receive other drugs as well, thus it is important to evaluate the association of thrombocytopenia with various other drugs which could attribute to its etiology.

The parenteral glycoprotein IIb/IIIa receptor inhibitors (GPIs) are common among these drugs, which have been known to be associated with thrombocytopenia [5]. During percutaneous coronary interventions (PCIs) and in the management of patients with the acute coronary syndrome, GPIs are among the widely used drugs that act on platelet glycoprotein IIb/IIIa receptors thereby blocking the fibrinogen binding and preventing thrombus formation by inhibiting platelet to platelet aggregation [6].

Various studies have shown few typical patterns of GPI-induced thrombocytopenia, some of which include acute pattern which manifests within 12 hours from the first exposure, acute pattern which manifests within 12 hours of a second exposure, the delayed-type of thrombocytopenia which manifests in five to seven days of treatment, platelet clumping resulting in pseudo-thrombocytopenia, and the variant presenting as anaphylaxis secondary to exposure [7]. Considering the pathophysiology behind the thrombocytopenia induced by glycoprotein IIb IIIa receptor antagonists (GPRAs), various hypotheses have been proposed. One of them being that, GPRAs lead to a conformational change in the GP IIb/IIIa receptors on the surface of the platelets, which leads to the formation of new epitopes that when recognized by preexisting antibodies in the plasma result in the coating of platelets and subsequently their removal from the circulation [8]. Sometimes platelet-bound drugs may persist in the circulation for weeks post GPRA treatment. These drug-bound platelets when coming in contact with newly formed antibodies are destroyed and removed from the circulation thereby responsible for delayed onset thrombocytopenia [9]. Various studies in the past have shown that higher incidence of thrombocytopenia has been seen with abciximab (2.4%) as compared to tirofiban (0.5%), but results are affected by whether the agent is an antibody or a synthetic compound, peptide or nonpeptide, the dose and duration of the drug used [10]. Close monitoring of platelet count is essential after initiation of GPRA treatment. The majority of cases with acute thrombocytopenia can be detected with close monitoring at 6, 12, and 24 hours post-GPRA treatment.

A study by Merlini et al. compared abciximab with tirofiban and confirmed that abciximab during PCI is associated with a higher incidence of thrombocytopenia [5]. The difference observed with abciximab and tirofiban may be due to the new epitopes generated by both of these drugs, which are more frequent with abciximab, which is an antibody derived and has more antigenicity than tirofiban which is a small peptide molecule.

In another study by Christen et al., a patient who had an inferior wall MI and underwent PCI developed thrombocytopenia, secondary to tirofiban. This patient developed shortness of breath and was subsequently diagnosed to have a diffuse alveolar hemorrhage [11].

In the EPIC study, patients who developed GPRAs-induced thrombocytopenia were shown to be at high risk of mortality and revascularization [12]. In the Platelet Receptor Inhibition for Ischemic syndrome management (PRISM) study, tirofiban was associated with a higher incidence of thrombocytopenia than heparin (1.1 vs 0.4%) [13].

In our patient, platelets started dropping around 8 h after tirofiban infusion. He had 208,000/microliter platelets on the day of admission, and the platelets decreased to 4000/microliter on day two. The possibility of HIT was kept but the workup for HIT was negative. Also, the disseminated intravascular coagulation (DIC) workup was negative. There was no evidence of schistocytes on peripheral smear. Hematology recommendations were sought, and one unit of platelets was transfused. Tirofiban infusion was stopped and platelets started rising after stopping the infusion.

HIT usually presents after five days in patients who have not been exposed to heparin previously or within minutes to hours in those who have had a prior exposure in six months [4]. Our patient had received aspirin and ticagrelor, but profound isolated thrombocytopenia has rarely been associated with these drugs [14]. The cases where isolated thrombocytopenia has been seen with these drugs are those that have been priorly sensitized or exposed to antibodies at the time of previous management with these drugs. It is seen in previous studies that clopidogrel led to thrombotic thrombocytopenic purpura however isolated thrombocytopenia is a rare occurrence [15].On discontinuation of the causative drug, in the case of drug-induced thrombocytopenia, the platelet count can return to the normal range in approximately seven to eight days [16]. Considering the clinical scenario in this patient, tirofiban-induced thrombocytopenia was more likely and our hypothesis was supported by the fact that on discontinuation of tirofiban treatment platelet count rapidly improved. Clofent-Sanchez et al. validated their hypothesis that tirofiban led to profound drug-induced thrombocytopenia by confirming the presence of antibodies using enzyme-linked immunoassay (ELISA) and flow cytometry [17]. 

The management of patients with thrombocytopenia in the setting of tirofiban infusion is tricky. Platelets should be strictly monitored in patients on GPRA, and these should be discontinued when platelet count drops below 100,000/microliter. In case of active bleeding, the patient should be treated with platelet and erythrocyte transfusions. If the hematocrit is >25% or hemoglobin levels >7 g/dL, blood transfusion should not be given, especially if the patient is hemodynamically stable because it may have a negative impact on the patients' outcome as it can lead to stent thrombosis. If the patient is hemodynamically unstable, patients or hemoglobin is <7 g/dL, there are no restrictions for blood transfusion. Intravenous immune globulin (IVIG) treatment (400 mg/kg/ day for 5 days) also can be used in the case of tirofiban-induced thrombocytopenia, especially if the patient is actively bleeding. For the patients without active bleeding, platelet transfusion with or without fresh frozen plasma or cryoprecipitate is indicated only for the patients with platelet count lower than 10,000/microliter [18].

## Conclusions

GP IIb/IIIa inhibitor-induced thrombocytopenia should be managed carefully. Because even though the bleeding after thrombocytopenia increases mortality rates of the ACS patients, the treatment of the bleeding with hemostatic agents increases the risk for reinfarction or stent thrombosis. Platelets should be carefully and routinely monitored in any patient who is on GPIs. 
